# Broiler litter moisture and trace metals contribute to the persistence of *Salmonella* strains that harbor large plasmids carrying siderophores

**DOI:** 10.1128/aem.01388-24

**Published:** 2025-03-13

**Authors:** Adelumola Oladeinde, Taejung Chung, Connie Mou, Michael J. Rothrock, Guoming Li, Ardeshir Adeli, Torey Looft, Reed Woyda, Zaid Abdo, Jodie Plumblee Lawrence, Denice Cudnik, Gregory Zock, Jose Teran, Xiang Li

**Affiliations:** 1 U.S. National Poultry Research Center, USDA-ARS 17123 , Athens, Georgia, USA; 2 SCINet Program, ARS AI Center of Excellence, Office of National Programs, USDA Agricultural Research Service 17123 , Beltsville, Maryland, USA; 3 Danisco Animal Nutrition & Health (IFF), Cedar Rapids, Iowa, USA; 4 Department of Poultry Science, University of Georgia 467744 , Athens, Georgia, USA; 5 Genetics and Sustainable Agriculture Research, USDA-ARS 17123 , Mississippi State, Mississippi, USA; 6 National Animal Disease Center, USDA-ARS 17123 , Ames, Iowa, USA; 7 Department of Microbiology, Immunology and Pathology, Colorado State University 164597 , Fort Collins, Colorado, USA; 8 Program of Cell and Molecular Biology, Colorado State University 3447 , Fort Collins, Colorado, USA; 9 College of Civil Engineering, University of Georgia 1355 , Athens, Georgia, USA; Anses, Maisons-Alfort Laboratory for Food Safety, Maisons-Alfort, France

**Keywords:** *Salmonella*, chicken, broiler litter, siderophores, food safety

## Abstract

Broiler litter sampling has proven to be an effective method for determining the *Salmonella* status of a broiler chicken flock and understanding the ecology of *Salmonella* prior to harvest. In this study, we investigated the ecology of *Salmonella* within the litter (*n* = 224) from two commercial broiler houses in the United States. We employed culture enrichment methods and quantitative polymerase chain reaction to determine the prevalence and load of *Salmonella* and utilized antimicrobial susceptibility testing and whole-genome sequencing (WGS) to characterize select isolates. Additionally, we applied machine learning algorithms and *in vitro* experiments to identify environmental selective pressures that may contribute to the persistence of *Salmonella* in litter. Our findings indicate that the prevalence and abundance of *Salmonella* in broiler litter are influenced by the downtime between flocks as well as by the flock raised on the litter. A Decision Tree Classifier model developed demonstrated that the moisture in the caked part of litter was the most influential environmental parameter for predicting the prevalence of viable *Salmonella*. WGS analysis revealed that Typhimurium, Infantis, and Kentucky strains that harbored large self-conjugative plasmids encoding fitness factors for iron siderophore production were the dominant *Salmonella* population found in litter, and exposure to iron-limiting and copper-enriched culture media affected *Salmonella* growth. Our results suggest that trace metals may select for siderophores harbored on plasmids, and interventions that reduce litter moisture can potentially curtail the persistence of *Salmonella* in pre-harvest environments.

**IMPORTANCE:**

Broiler chicken meat is the most consumed protein worldwide, and global poultry imports are projected to reach 17.5 million tons by 2031. To raise billions of chickens, litter is reused multiple times by the top global producers and exporters of chicken (Brazil and the United States). Chickens are in continuous contact with litter and depend on it for warmth and coprophagy. Consequently, litter serves as a major route for pathogens such as *Salmonella* to infect chickens, making it crucial to understand the environmental and genetic selective pressures that might explain why certain *Salmonella* strains persist on broiler farms more than others. In this study, we demonstrated that *Salmonella* strains that harbored siderophores on large conjugative plasmids persisted in litter and suggested that reducing litter moisture would significantly control *Salmonella* prevalence. However, a complete eradication of persisting *Salmonella* strains will require novel, innovative, and multifaceted approaches.

## INTRODUCTION

*Salmonella* infections are commonly associated with poultry consumption. Although the number of chicken samples testing positive for *Salmonella* after harvest has significantly decreased since the implementation of Key Performance Indicators in the United States, the overall incidence of salmonellosis remains unchanged (1–4). To achieve the Healthy People goal of a 25% reduction in *Salmonella* food-borne illnesses by 2030 (https://health.gov/healthypeople), novel, practical, and efficient food safety approaches will be required. The United States Food Safety and Inspection Service (FSIS) proposed regulatory framework for *Salmonella* control emphasizes *Salmonella* load reduction at pre-harvest. This includes the requirement that incoming flocks be tested for *Salmonella* load and serotype before entering a processing establishment (5). However, there are no methods that can accurately determine the load of *Salmonella* in carcasses or environmental matrices such as feces or litter. Furthermore, there are insufficient data to indicate that *Salmonella* serotypes and strains recovered at pre-harvest are identical to strains found in post-harvest.

Broiler chicken litter (a mixture of decomposed wood shavings, rice hulls, or sawdust mixed with chicken feces, uric acid, feed, and other broiler-sourced materials [6]) sampling has been shown to be a practical approach for detecting a *Salmonella*-infected flock at pre-harvest (7). Research has shown that the presence of *Salmonella* in the litter before chickens are placed on it and during harvesting is a key predictor of broiler chicken carcass *Salmonella* status (8, 9). Countries like the United States and Brazil often reuse litter to raise multiple flocks of broiler chickens (10–12). Between flocks, litter management practices include decaking, windrowing, or acidification to reduce ammonia and pathogens (13–15). In the long term, litter is applied to soils as biological soil amendment of animal origin or sold as fertilizers (16). Studies on the occurrence of *Salmonella* in litter have shown that *Salmonella* prevalence may decrease over successive use of litter but can persist and re-infect incoming flocks (10, 12, 17–19). Roll et al. (12) found that the number of litter samples positive for *Salmonella* decreased over 14 consecutive flocks and concluded that the more times the litter is reused, the lower the number of *Salmonella*-positive litter samples. Voss-Rech et al. (17) showed that *Salmonella* Heidelberg persisted in litter throughout the grow-out of six consecutive flocks, while Sevilla-Navarro et al. (20) demonstrated that *Salmonella* Infantis persisted in multiple broiler houses even after multiple rounds of cleaning and disinfection. Using Bayesian models, Machado Junior et al. (10) predicted that the bactericidal effect of litter diminishes after it has been reused to grow six consecutive broiler flocks. Our group has previously shown that *Salmonella* prevalence in litter decreased during the grow-out of three consecutive flocks (21). These earlier studies revealed the inherent complexities of detecting *Salmonella* in litter but also reaffirmed the notion that *Salmonella* can persist in a broiler house over time and can become a source of re-infection during the grow-out of broiler chickens.

In recent studies, serovars Kentucky, Infantis, Typhimurium, and Enteritidis were the most common serovars found in broiler litter sampled across a few states in the United States (22, 23). Whole-genome sequencing of *Salmonella* isolates found in litter has revealed that they harbor transferable mobile DNA (e.g., plasmids) that often carry genes conferring resistance to antibiotics, metals, and quaternary ammonium compounds (24–27). While current findings have improved our understanding of “which salmonellae are present” and the genes they carry, we still have limited insight into the environmental and genetic selective pressures that might explain why certain *Salmonella* strains persist on broiler farms more than others.

In this study, we sampled the litter of two commercial broiler houses co-located on one farm over four successive flocks. Our objectives were to determine the prevalence, abundance, and serotypes of *Salmonella* in litter, identify strain-level genotypes and phenotypes, and investigate litter environmental properties that may explain their persistence in litter.

## RESULTS

### *Salmonella* prevalence and abundance in litter

**Fig 1:**
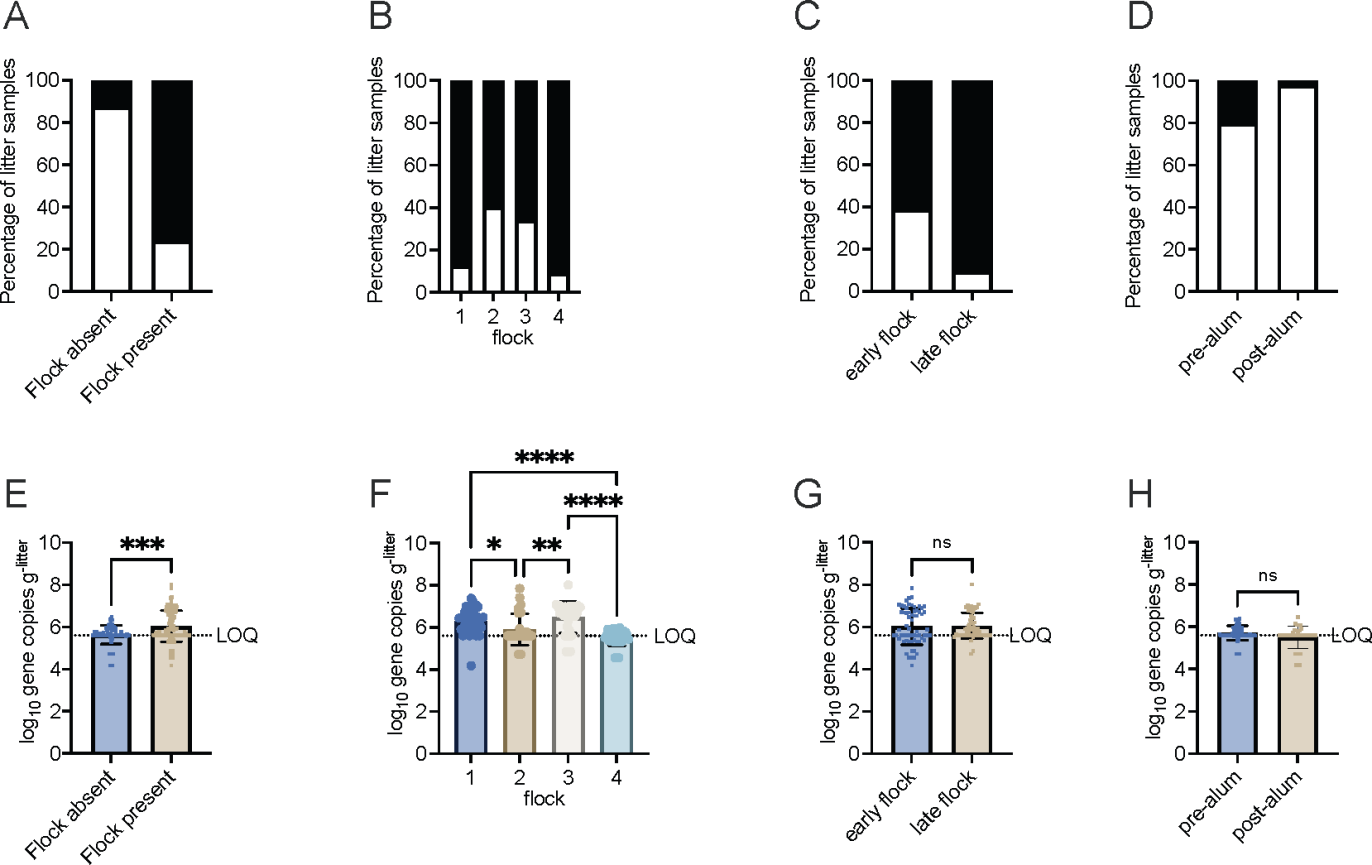
Culturable *Salmonella* prevalence and gene abundance in litter samples. Prevalence (A–D) and *ttr* gene abundance (E–F) were grouped by the presence of a chicken flock/during downtime of a chicken flock (**A and E**), the flock (**B and F**), the flock status (early: 4–14 days old and late: 32–38 days old) (**C and G**), and downtime (**D and H**). A–D: *Salmonella* present, black bar; *Salmonella* absent, white bar; E–F: Mann-Whitney test; *P < 0.05, **P < 0.01, ***P < 0.001, and ****P < 0.0001. LOQ, limit of quantitation.

Overall, *Salmonella* prevalence in litter samples was 48.6% (*n* = 224). There was no significant difference in prevalence (Fisher’s exact test; *P* = 0.35) between house 2 and house 4. *Salmonella* prevalence was significantly different between samples collected when chickens were absent (downtime) and when they were present (grow-out) (*P* < 0.05). Likewise, the flock raised and broiler age at the time of sampling significantly affected *Salmonella* prevalence (*P* < 0.05) (Fig. 1A through D). During grow-out, *Salmonella* prevalence was 76%, while it was 12.5% during downtime (Fig. 1A). Litter collected during the grow-out of flock 2 and flock 3 had lower prevalence than litter from flocks 1 and 4 (Fig. 1B). Furthermore, litter samples collected when chickens were older or at market age (late flock) tended to have a higher prevalence of *Salmonella* compared to litter collected when chickens were young (early flock) (*P* = 0.0001; Fig. 1C). During downtime, *Salmonella* prevalence was lower in litter collected after alum was applied (2.5%) compared to samples collected before alum application (19.6%) (*P* = 0.012; Fig. 1D).

To determine *Salmonella* abundance, we used quantitative PCR (qPCR) primers targeting tetrathionate reductase (*ttrBCD*) locus (28). *Salmonella* abundance ranged from <log_10_ 5.60–log_10_ 8.01 gene copies g^-litter^ (mean = log_10_ 5.86 ± 0.66 gene copies g^-litter^). Like prevalence, there was no significant difference in abundance between house 2 and house 4 (Mann-Whitney test; *P* = 0.3416). *Salmonella* load in litter was significantly different between downtime and grow-out samples and between flocks (*P* = 0.0002). During downtime (*n* = 96), average *Salmonella* abundance was lower (log_10_ 5.63 ± 0.44 gene copies g^-litter^) compared to during grow-out (*n* = 128; log_10_ 6.04 ± 0.74 gene copies/g^-litter^) (Fig. 1E). *Salmonella* load in litter collected from flocks 1 and 3 was higher than in flocks 2 and 4 (*P* < 0.05) (Fig. 1F). In contrast to *Salmonella* prevalence, there was no difference (*P* = 0.49) in *Salmonella* gene abundance between litter samples collected when chickens were younger versus older (Fig. 1G). Likewise, during downtime, there was no difference in abundance between litter samples collected before and after alum application (*P* = 0.14; Fig. 1H). This result suggested that *Salmonella* prevalence and abundance in litter were mostly influenced by the downtime between flocks and the broiler flock in the house.

### *Salmonella* serotypes and antibiotic resistance profile

**Fig 2:**
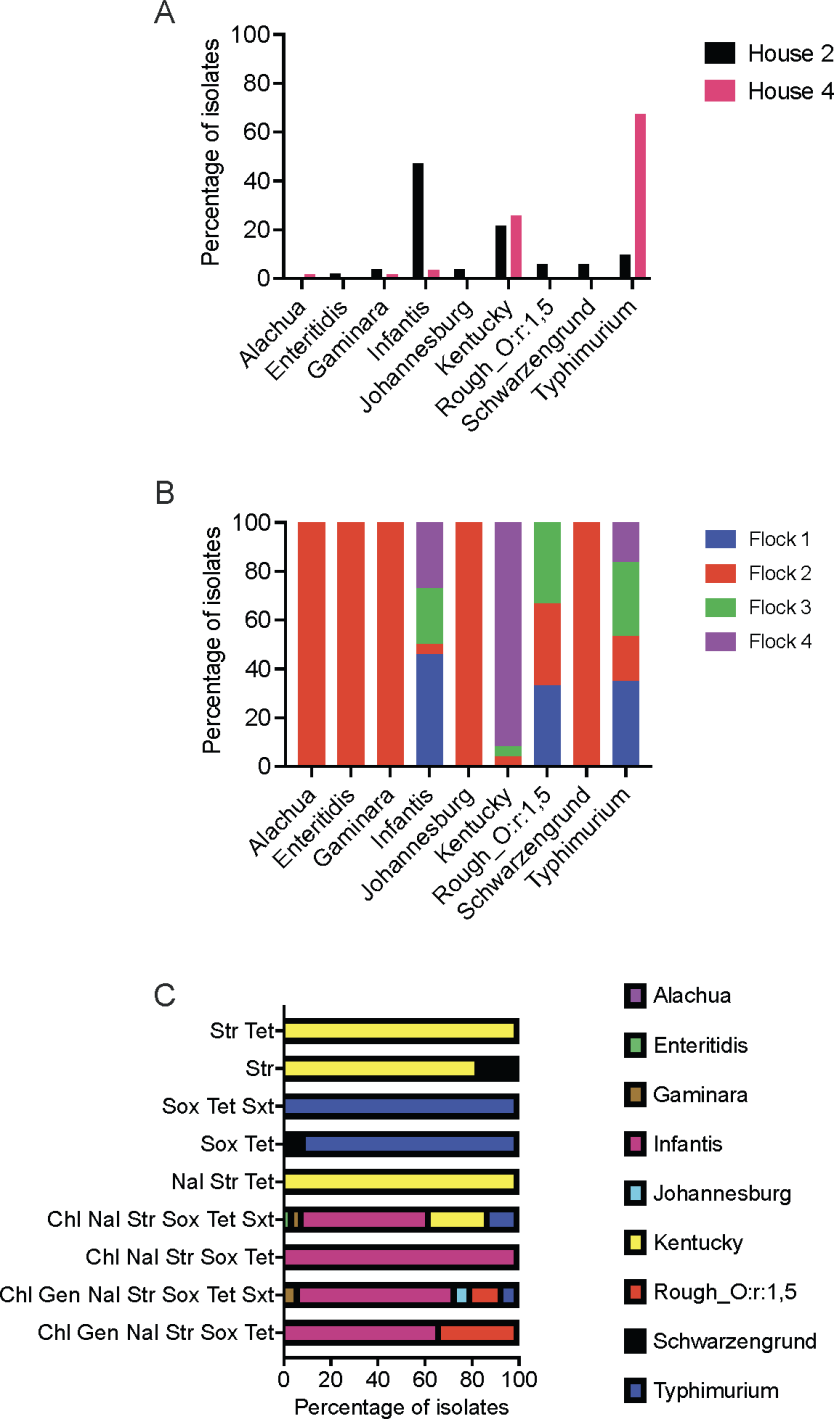
*Salmonella* serotypes found in litter and their antibiotic resistance profile. *Salmonella* serotypes were grouped by (A) house, (B) flock and (C) antibiotic resistance profile. Chl, chloramphenicol; Gen, gentamicin; Nal, nalidixic acid; Str, streptomycin, Sox, sulfisoxazole; Tet, tetracycline; Sxt, trimethoprim-sulfamethoxazole.

Traditional serotyping using antisera and antibiotic susceptibility via broth microdilution was performed on one randomly selected isolate from each *Salmonella* positive litter sample (*n* = 109). The isolates were classified into nine serotypes, including Typhimurium (*n* = 44), Kentucky (*n* = 26), and Infantis (*n* = 26), which made up the majority of isolates. There was a significant difference in serovars found between houses (Fisher’s exact test; *P* < 0.0001). We isolated Infantis more frequently from the litter samples collected from house 2 compared to house 4, while Typhimurium were isolated more often from house 4 (Fig. 2A). *Salmonella* isolates from house 2 (*n* = 51) were grouped into eight serotypes, while house 4 isolates (*n* = 58) belonged to five serotypes. Typhimurium and Infantis were isolated from the litter of each broiler flock cohort (Fig. 2B). Flock 4 litter samples more frequently harbored serovar Kentucky (22/24), while flock 2 samples harbored at least one isolate that belonged to each of the nine serovars (Fig. 2B). During downtime, only 1 post-alum sample was positive for *Salmonella,* while 11 pre-alum samples were *Salmonella* positive. Schwarzengrund was the serotype found in the post-alum sample, while serovars Typhimurium (*n* = 5), Kentucky (*n* = 3), and Infantis (*n* = 2) were isolated sporadically pre-alum application (Table S1).

Infantis and Typhimurium were the dominant serovars found in litter, and Infantis isolates were resistant to multiple classes of antibiotics. Infantis isolates were resistant to five to seven antibiotics, including chloramphenicol, nalidixic acid, streptomycin, sulfisoxazole, and tetracycline, while Typhimurium isolates were mostly resistant to sulfisoxazole and tetracycline (Fig. 2C; Fig. S1). Kentucky isolates were resistant to streptomycin (Fig. 2C; Fig. S1). Seventeen isolates serotyped as Kentucky (*n* = 6), Typhimurium (*n* = 4), rough_O:r:1,5 (*n* = 3), Gaminara (*n* = 2), Enteritidis (*n* = 1), and Johannesburg (*n* = 1) exhibited the same resistance pattern as Infantis. In addition, the distance matrix and hierarchical clustering analyses grouped these isolates into the same clusters as the Infantis isolates (Fig. S1). To ensure that the incongruence in resistance pattern was not due to serotype misclassification using antisera, we used qPCR primers specific for Infantis (29) for further confirmation. qPCR results suggested that 11/17 isolates were Infantis (Tables S1 and S2). A second susceptibility testing of a few Typhimurium and Kentucky isolates that showed a similar resistance profile as Infantis indicated that they were not multidrug resistant (resistance to three or more antimicrobial drug classes) but were resistant to either sulfisoxazole and tetracycline or streptomycin (Table S2). One possible explanation for the inconsistent antibiotic susceptibility results is that the antimicrobial resistance genes (ARGs) that confer the relevant resistance phenotypes are located on mobile genetic elements that can be lost or gained, such as plasmids and transposons.

### Genome diversity of *Salmonella* isolates

**Fig 3:**
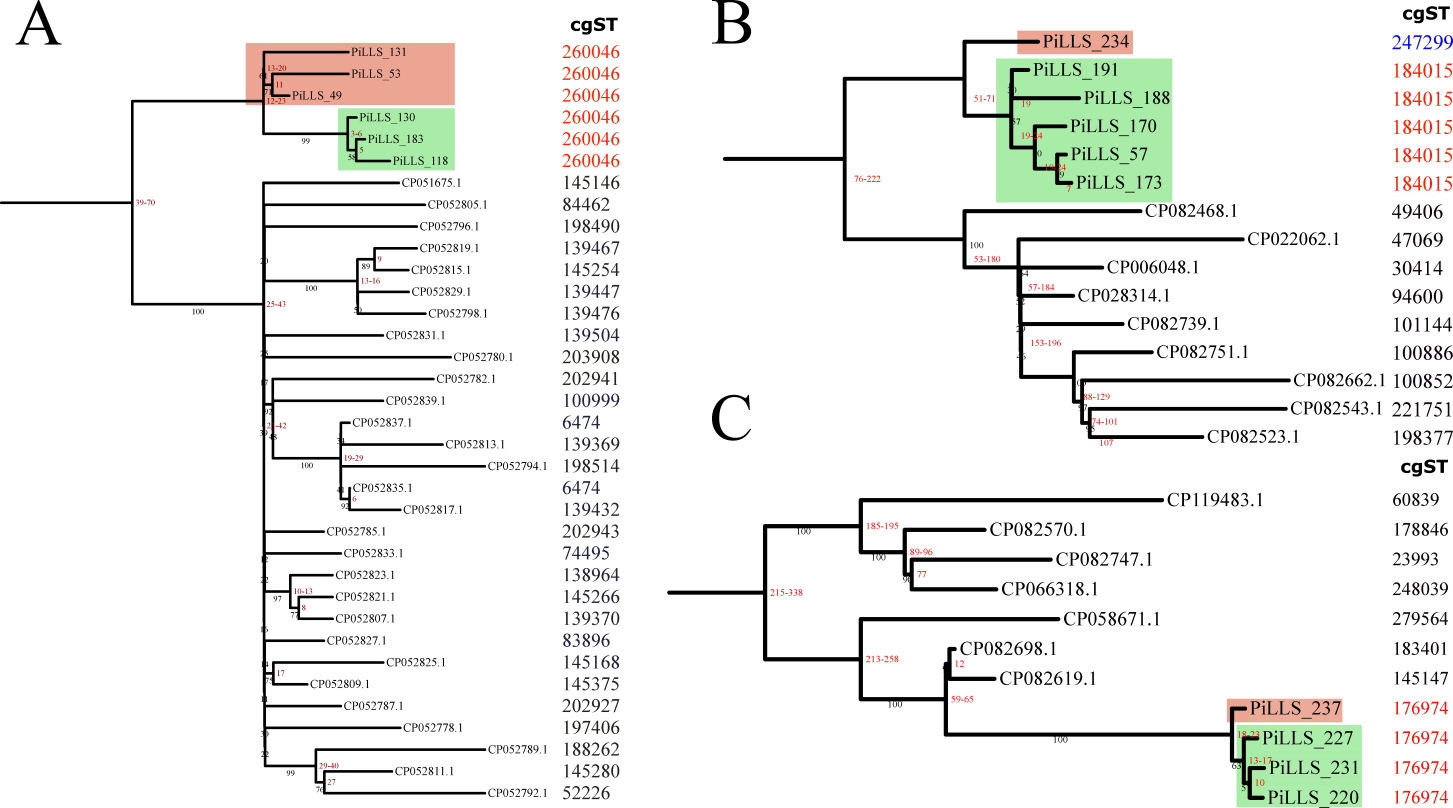
SNP-based maximum-likelihood phylogenetic trees for chromosomes of three different *Salmonella* serotypes: (**A**) Infantis, (**B**) Typhimurium, and (**C**) Kentucky. Bootstrap values are shown in black, and SNP distance of each clade is shown in red. Adjacent to the tips of the tree are the cgMLST sequence types (STs) for each strain (blue- and red-colored cgST numbers indicate strains from this study). Outgroups are complete Infantis genomes from Tyson et al. (30) and complete Typhimurium and Kentucky genomes found in the NCBI database that harbored IncC and IncH1B plasmids that were identical to the ones in this study.

Long and short read sequencing was performed on 23 *Salmonella* isolates. These isolates were selected based on serotype and AST profile. Table S2 shows the metadata associated with each sequenced genome. Nine isolates classified by antisera-based serotyping (Enteritidis [*n* = 1], Kentucky [*n* = 2], Gaminara [*n* = 2], Johannesburg [*n* = 1], and Rough_O:r:1,5 [*n* = 3]) were confirmed by WGS to be Infantis (Table S2). Consequently, Infantis was the major serovar sequenced (13/23), followed by Typhimurium (6/23) and Kentucky (4/23). Infantis, Typhimurium, and Kentucky isolates were classified into multilocus sequence types (MLSTs) 32, 19, and 152, respectively. A core genome MLST (cgST) categorized all Infantis isolates as cgST 260046 and five Typhimurium isolates as cgST 184015. One Typhimurium isolate (PiLLS-234) was categorized as cgST 247299, while all Kentucky isolates were cgST 176974. The maximum number of single nucleotide polymorphism (SNP) differences between Infantis isolates was 22 (median = 11, range = 1–22), while Typhimurium isolates differed by a maximum of 71 (median = 21, range = 7–71) (Fig. 3A through C). Kentucky isolates differed from each other by a maximum of 23 SNPs (median = 17.5, range = 10–23) (Fig. 3C). A phylogenetic tree reconstructed using the SNPs grouped isolates from each serovar (Infantis, Typhimurium, and Kentucky) into two clades (Fig. 3A through C).

### Antimicrobial resistance determinants found in *Salmonella* genomes

**Fig 4:**
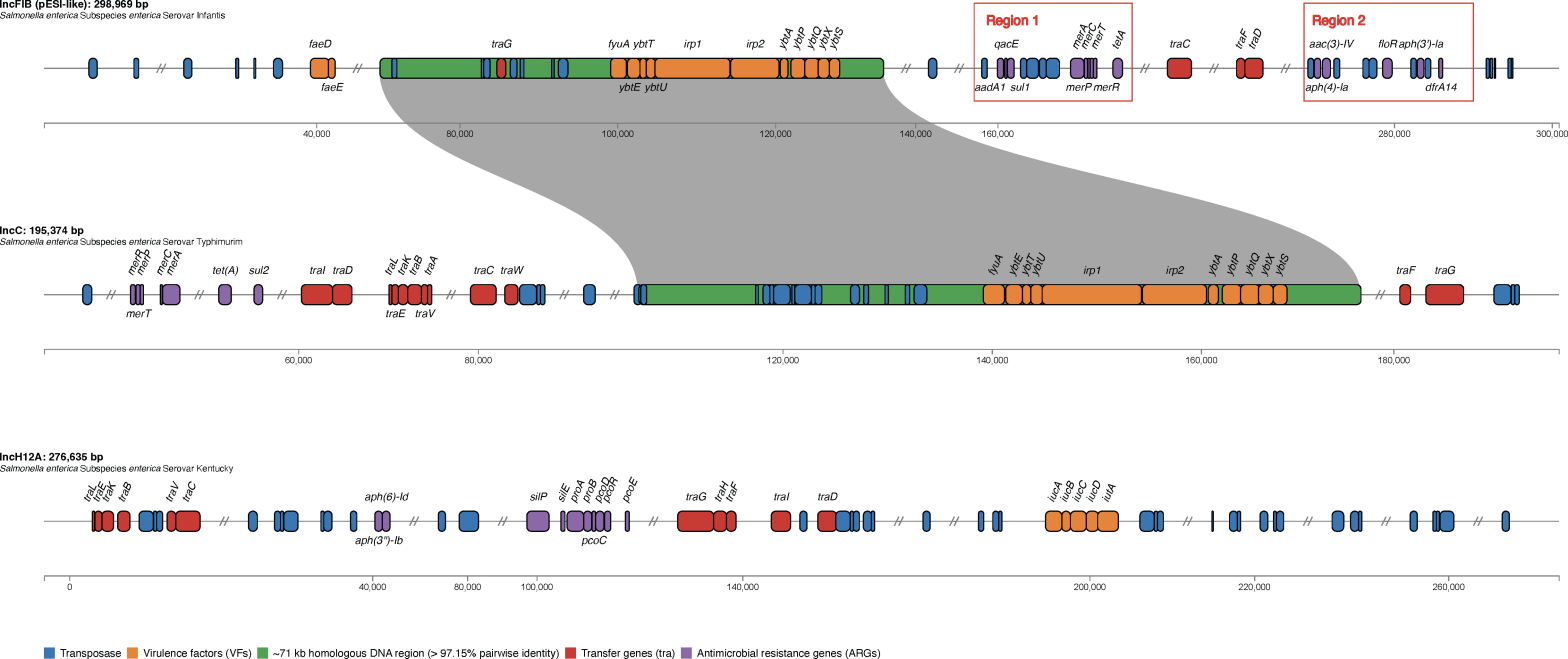
Comparative map of three representative plasmids from three different serotypes: *S*. Infantis (IncFIB [pESI-like]), *S*. Typhimurium (IncC), and *S*. Kentucky (IncH12A). Identified antimicrobial resistance genes (purple), virulence factors (orange), transposases (blue), and transfer genes (red) are highlighted. The green box shows ~71 kb homologous region between the IncFIB and IncC plasmids, containing *Yersinia* high pathogenicity island.

We asked if isolates differed in their carriage of ARGs and plasmids. Infantis isolates harbored seven to nine ARGs, including a chromosomal mutation in *gyrA* (D87Y) that conferred resistance to nalidixic acid in all isolates. ARGs [*aadA1, aac(3)-IV, aph(3′)-Ia, aph(4)-Ia, dfrA14, floR, sul1,* and *tetA*] were found on one plasmid. Typhimurium isolates (5/6) harbored two ARGs (*sul2* and *tetA),* while one isolate carried five ARGs [*aadA1, aac(3)-IV, dfrA1, sul2,* and *tetA*]. All ARGs found in Typhimurium were located on plasmids (Fig. 4; Fig. S2). All four Kentucky genomes harbored two ARGs [*aph(3″)-Ib* and *aph (6)-Id*] on one plasmid (Fig. 4).

Infantis harbored one large plasmid [IncFIB(pN55391)] average of 306,088 bp (SD = 665.48 bp) (also known as the plasmid of Emerging *Salmonella* Infantis like [pESI-like]), while Typhimurium and Kentucky harbored two to five plasmids. All four Kentucky isolates harbored plasmid IncHI2A (276,635 bp, untypeable pMLST) and IncX1 (51,712 bp), while five Typhimurium isolates carried IncC (195,374 bp [SD = 0], pMLST 2), IncX1 (34,694 bp [SD = 0]), ColRNAi (4,681 bp [SD = 4.9 bp]), and Col(pHAD28) (3,609 bp [SD = 0]) (Fig. S2). Typhimurium isolate PiLLS-234 harbored IncC (176,922 bp), IncI1 (pMLST 12), IncX1, Col8282, and ColpVC (Fig. S2). As expected, all ARGs found in Infantis were carried on the pESI-like IncFIB plasmid, while ARGs found in Kentucky isolates were harbored on IncH12A plasmid (Fig. 4). Additionally, genes predicted to confer resistance to mercury (*merRTPCA*) and quaternary ammonium (*qacE*) were harbored on a pESI-like IncFIB plasmid, while IncH12A carried genes for copper (*pcoABCDRE*) and silver (*silP* and *silE*) resistance (Fig. 4). In all Typhimurium isolates, IncC plasmids carried *sul2*, *tetA,* and *merRTPCA*, while IncI1 harbored *aadA1, aac(3)-IV,* and *dfrA1* (Fig. S2).

### Genetic context of transmissible fitness factors found in *Salmonella* genomes

We found 117, 118, and 109 virulence genes (VGs) in Infantis, Typhimurium, and Kentucky isolates, respectively. All three serovars had 99 VGs categorized into five virulence/fitness classes, including fimbrial adherence determinants, secretion systems (*Salmonella* pathogenicity islands 1 and 2), and magnesium uptake. VGs encoding the siderophore yersiniabactin (*ybtETUAPQXS*, *fyuA*, *irp1,* and *irp2*) were unique to both Infantis and Typhimurium, while VGs for siderophore aerobactin (*iucABCD* and *iutA*) were found in Kentucky isolates (Fig. 4). The *Yersinia* high pathogenicity island (HPI) was carried on the pESI-like IncFIB and IncC plasmids, while genes for aerobactin synthesis were on IncH12A plasmid (Fig. 4).

Yersinibactin (Ybt) is a siderophore that has a high binding affinity for ferric and cupric ions (31). Enterobacteriaceae that carry Ybt have increased fitness in the environment and a higher ability to cause virulent diseases (32, 33). pESI-like IncFIB plasmids that carry Ybt have been reported; however, Ybt occurrence in Typhimurium isolates carrying IncC plasmids has not been reported. To evaluate their similarity and genetic structure, we compared the genome sequences of pESI-like IncFIB and IncC plasmids from this study. Pairwise DNA alignment and whole plasmid comparison indicated that the plasmids are 32%–33% identical and share a ~71 kb homologous DNA region (>97.15% pairwise identity) encoding Yersinia HPI (Fig. 4). The flanking coding DNA sequences of the region were highly variable and encoded for transposases, tyrosine-type recombinase/integrase, DNA replication terminus binding site, and hypothetical proteins.

### *Salmonella* prediction using machine learning

To determine the litter bacterial population and physico-chemical parameters affecting *Salmonella* prevalence on farms, we used machine learning (ML) techniques. Seven ML classifiers were utilized to predict if a litter sample would test positive or negative for *Salmonella* culture. The bacterial variables used were qPCR-based gene abundance results for *Salmonella enterica* (*ttr,* log_10_ 4.18–8.01), *S*. Infantis (*usg*, log_10_ 4.56–8.37), total Enterobacteriaceae (*gapA,* log_10_ 4.64–10.43), the ratio of *Salmonella* to total Enterobacteriaceae (*ttr*/*gapA*, −5.39–1.45), and the ratio of *S*. Infantis to total Enterobacteriaceae (*usg*/*gapA*, −5.23–3.04). *S*. Infantis was included due to its prevalence as a major serotype isolated from litter in this study and its current global spread (19, 20, 30, 34). Environmental variables used were litter moisture, both the caked (14.6%–65.5%) and friable/loose (14.0%–48.2%) portions and litter pH, which ranged from 3.2 to 7.7. A comparative evaluation determined that the Decision Tree Classifier was the most effective, with a weighted average accuracy of 96% (Table 1). The top-ranked variables influencing the ML model’s predictions were the moisture level in the caked part of the litter, the *ttr*/*gapA* ratio, and *gapA* abundance (Table 2).

**TABLE 1:** Testing performance of machine learning classifiers for *Salmonella* prevalence prediction

Model name	Optimal hyperparameters	Precision	Recall	F1 score	Accuracy
Adaptive Boosting Classifier	learning_rate: 0.1, n_estimators: 100	0.90	0.87	0.87	0.87
Decision Tree Classifier	criterion: “gini,” max_depth: None, min_samples_leaf: 2, min_samples_split: 5	0.96	0.96	0.96	0.96
Gaussian Naive Bayes	var_smoothing: 1 × 10^–9^	0.87	0.87	0.87	0.87
Logistic Regression	C: 100, solver: “newton-cg”	0.84	0.83	0.83	0.83
Multi-layer Perceptron Classifier	activation: “tanh,” alpha: 0.0001, hidden_layer_sizes: (100, 50, 50), learning_rate: “constant,” solver: “adam”	0.37	0.61	0.46	0.61
Random Forest Classifier	max_depth: None, max_features: “log2”, min_samples_split: 10, n_estimators: 100	0.87	0.87	0.87	0.87
Stochastic Gradient Descent Classifier	alpha: 0.001, learning_rate: “optimal,” loss: “perceptron,” penalty: “l1”	0.37	0.61	0.46	0.61

**TABLE 2:** Ranking and feature importance score for different machine learning models^*a*^

Features or independent variables	Adaptive Boosting Classifier		Decision Tree Classifier		Gaussian Naive Bayes		Logistic Regression		Multi-layer Perceptron Classifier		Random Forest Classifier		Stochastic Gradient Descent Classifier	
	Ranking	FIS	Ranking	FIS	Ranking	FIS	Ranking	FIS	Ranking	FIS	Ranking	FIS	Ranking	FIS
*gapA*	6	0.10	3	0.14	1	0.59	8	0.10	6	10.47	8	0.09	8	4.05
*ttr*	7	0.08	6	0.02	4	0.40	3	0.84	7	9.89	4	0.11	2	18.56
*usg*	3	0.14	4	0.13	2	0.46	4	0.74	5	10.72	7	0.09	5	10.11
*ttr/gapA*	1	0.20	2	0.21	5	0.19	1	0.94	1	11.81	3	0.13	3	14.58
*usg/gapA*	4	0.12	5	0.08	6	0.13	5	0.64	8	9.68	5	0.11	4	14.11
Litter pH	8	0.08	8	0.01	3	0.44	2	0.92	3	10.88	6	0.10	6	8.21
Caked litter moisture	2	0.16	1	0.39	7	0.02	6	0.54	4	10.81	1	0.24	1	23.13
Friable litter moisture	5	0.12	7	0.02	8	0.02	7	0.14	2	11.28	2	0.13	7	5.86

^*a*^ FIS, feature importance score.

### Correlation between litter moisture and nutrients

The feature importance scoring is an impurity-based method (35) and is crucial for determining which features significantly influence *Salmonella* prediction. Feature importance scores may vary in scale due to different computational methods and model architectures (Table 2). Typically, a feature with a higher score has a greater impact on predicting *Salmonella* prevalence, while scores near zero suggest minimal or no relevance to the prediction. To understand the relationship between the top three variables and *Salmonella* detection, we analyzed their comparison across litter samples that were either positive or negative for culturable *Salmonella*.

**Fig 5:**
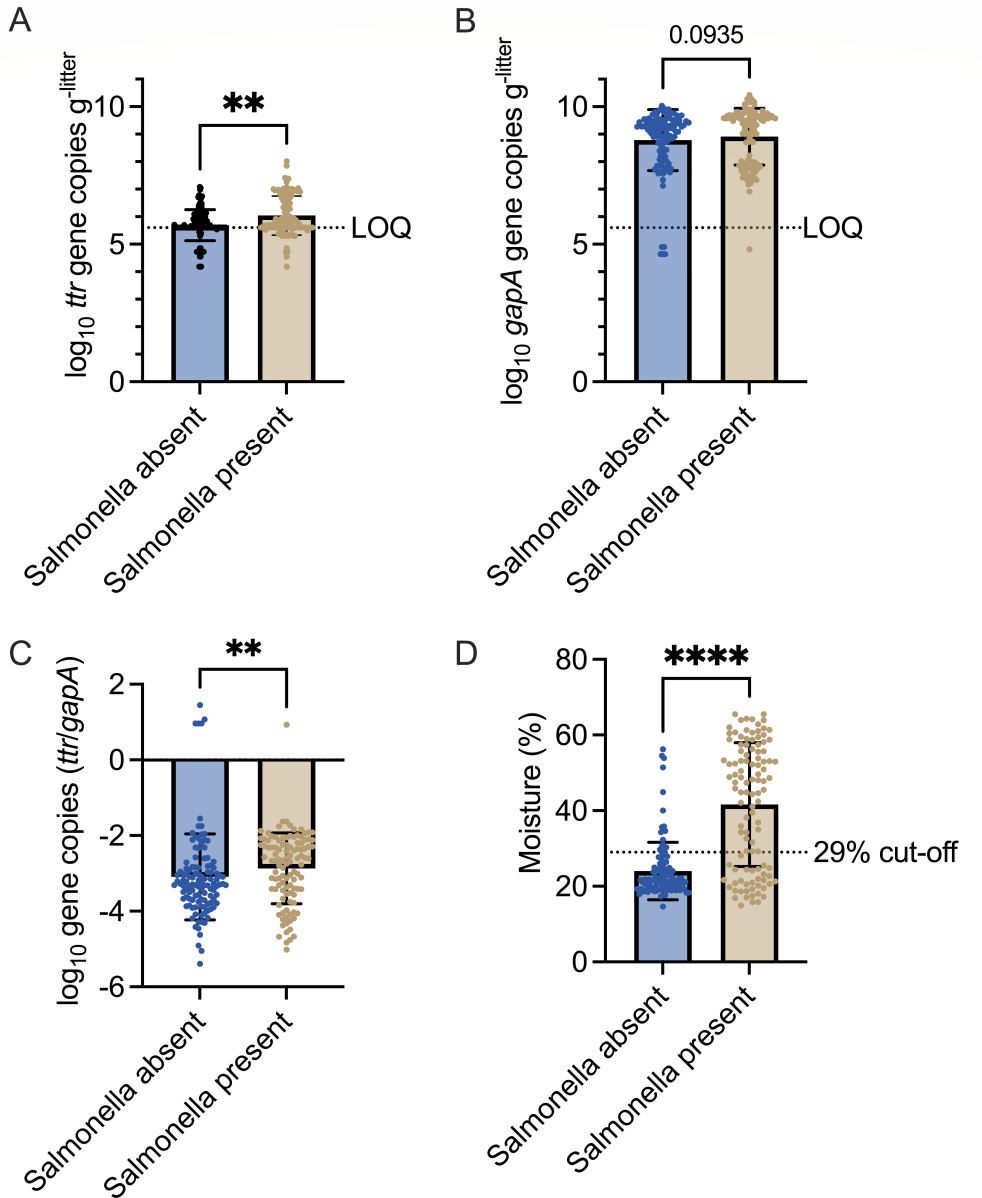
Most influential variables in the machine learning model. Abundance of *Salmonella ttrBCD* (**A**) and total Enterobacteriaceae *gapA* gene (**B**), ratio of *ttrBCD/gapA* (**C**), and percentage of moisture in caked part of litter (**D**).

Litter samples that were culture positive for *Salmonella* harbored significantly higher abundance of *Salmonella* (mean = log_10_ 6.04 ± 0.71 gene copies g^-litter^) than negative samples (log_10_ 5.69 ± 0.56 gene copies g^-litter^, Mann-Whitney test; *P* = 0.0025) (Fig. 5A). Similarly, total Enterobacteriaceae abundance and *Salmonella* relative abundance (*ttr*/*gapA*) were higher in *Salmonella* culture-positive than negative litter samples (Fig. 5B and C). Litter samples that were culture positive for *Salmonella* had higher caked litter moisture (mean = 41.6% ± 0.16%) than culture negative samples (mean = 24.0% ± 0.07%) (Fig. 5D) (Mann-Whitney test; *P* < 0.0001), and the caked portions of litter had higher moisture (mean = 32.6% ± 15.4%) than the friable parts (mean = 23.8% ± 6.2%). The odds of finding a litter sample that is culture positive for *Salmonella* increased by 24,920 (95% CI: 1,869–501,005) for every 10% increase in caked litter moisture (logistic regression; *P* < 0.0001). When the moisture in the caked parts of the litter was >29.0%, ~69% of litter samples were positive for *Salmonella* (Fig. 5D). Wet caked litter samples were collected around the drinkers.

To gain more insights into the role of litter moisture, we measured the levels of 13 nutrients in litter samples collected during the grow-out of one flock (early and late) and one downtime (pre- and post-alum application) (*n* = 63) (Table S1). We reasoned that trace nutrients are crucial for pathogen survival and that water is a major driver of biologically available nutrients. Therefore, significant changes in trace nutrients could influence *Salmonella* populations in litter. Phosphorus (P) and iron (Fe) concentrations were the most significant nutrients found to be correlated with moisture in both caked and friable litter (Spearman correlation, *P* < 0.0001 [Fig. S3]). Fe concentrations in litter decreased as moisture increased (caked, rho = −0.480, 95% CI = −0.654 to −0.256; friable, rho = −0.475 = −0.651 to −0.250), while P showed a positive correlation (caked, rho = 0.46, 95% CI = 0.23–0.64; friable, rho = −0.44 = 0.21–0.62). Copper was also positively correlated with litter moisture (caked and friable, rho = 0.29, *P* = 0.02) (Fig. S3).

Furthermore, *Salmonella* (rho = −0.212, *P* = 0.095) and total Enterobacteriaceae (rho = −0.387, *P* = 0.002) gene abundances were negatively correlated with Fe, while their relationship with Cu and P was not significant (*P* > 0.05). We found that litter samples that had lower than average Fe levels (<664 ppm) tended to be *Salmonella* culture positive and carried higher gene abundance of total Enterobacteriaceae than *Salmonella* culture-negative samples (Fig. S4). These results suggest that litter moisture influences the nutrient levels and the abundance of *Salmonella* and total Enterobacteriaceae and has the potential to be an indicator for *Salmonella* prevalence at pre-harvest.

### Exposure to iron and copper affected the fitness of *Salmonella*

**Fig 6:**
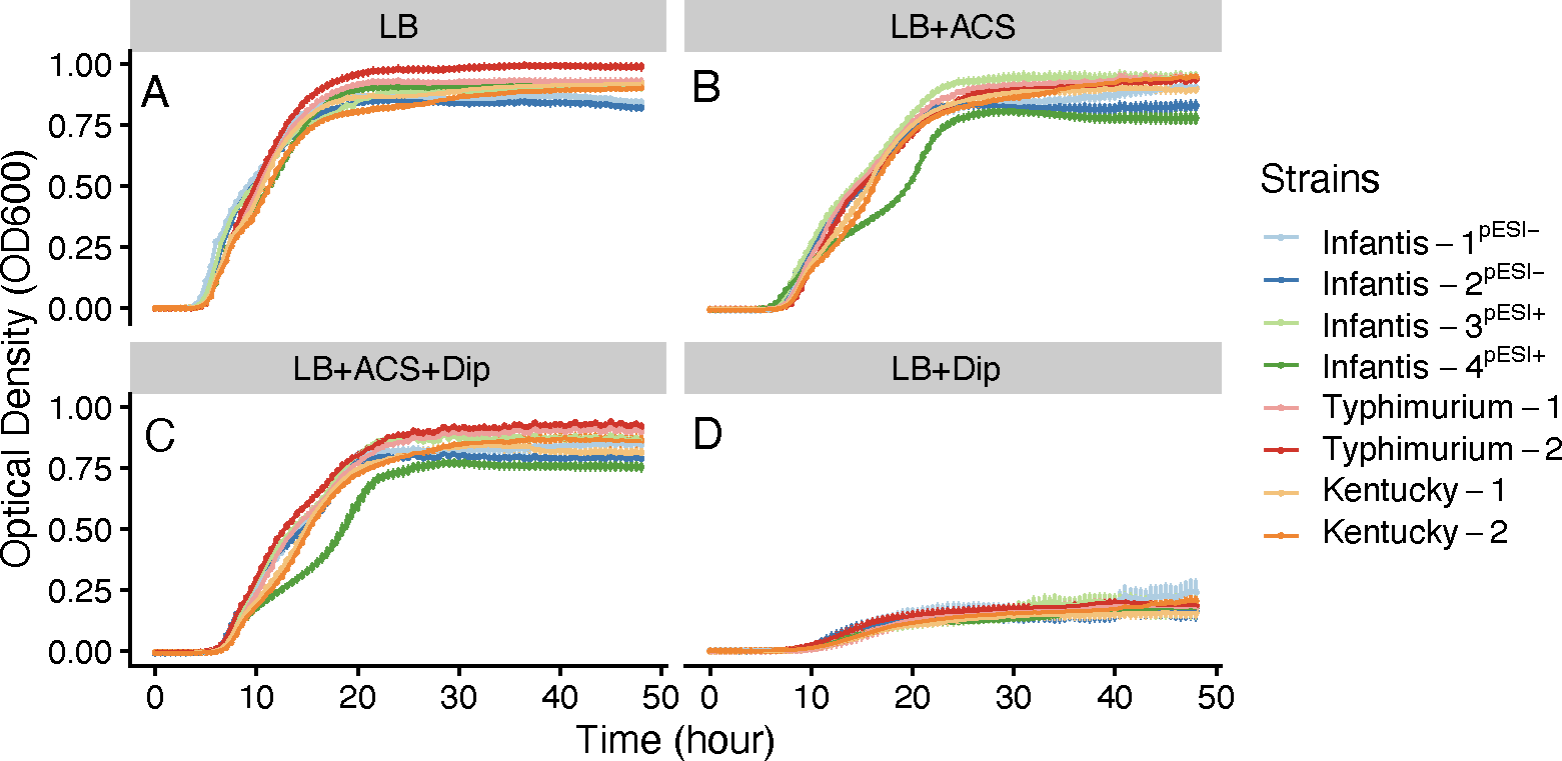
Changes in optical density (OD_600_) over 48 h of incubation in four different media: (**A**) lysogeny broth (LB), (**B**) LB with acidified CuSO_4_ (ACS) (LB + ACS), (**C**) LB with ACS and with an iron chelator (2,2-dipyridyl) (Dip) (LB + ACS + Dip), and (**D**) LB with Bipy (LB + Dip). Different colored lines represent eight selected *Salmonella* strains (two *S*. Infantis^pESI-^, two *S*. Infantis^pESI+^, two *S*. Typhimurium, and two *S*. Kentucky).

Iron and copper were among the significant variables correlated with litter moisture. Furthermore, the Infantis, Typhimurium, and Kentucky strains from this study carried large plasmids that encoded siderophore proteins that have a high affinity for metals such as iron and copper. Moreover, acidified CuSO_4_ (ACS) was routinely added to the drinking water of the broiler chickens in this study. Therefore, we explored the effect of iron limitation and copper enrichment on the growth of two randomly selected Infantis, Typhimurium, and Kentucky strains from this study. For comparison, we used two *S*. Infantis strains collected by FSIS in 2017 that do not carry the pESI-like plasmids and were pan-susceptible to all antibiotics tested, hereafter referred to as Infantis^pESI-^ (Table S3). First, we compared the maximum growth of each strain in lysogeny broth (LB). The two Typhimurium strains achieved higher growth in LB than Infantis^pESI-^ strains. (Kruskal-Wallis test, *P*.adj < 0.01) (Fig. 6A). Infantis strain 4^pESI+^ growth in LB was also higher than Infantis^pESI-^ strains (*P*.adj < 0.01), while Infantis strain 3^pESI+^ growth was only higher than Infantis-2^pESI-^ (*P*.adj < 0.05) (Fig. 6A). Kentucky strain 1 had higher growth in LB than Infantis-2^pESI-^ (*P*.adj < 0.01), but there was no difference between Kentucky strain 2 and Infantis^pESI-^ strains (*P*.adj > 0.05) (Fig. 6A).

In LB supplemented with ACS, Typhimurium strains achieved significantly higher maximum growth than Infantis-2^pESI-^ (*P*.adj < 0.05), while Typhimurium strain 1 was also higher than Infantis-1^pESI-^ (*P*.adj < 0.05) (Fig. 6B). Likewise, in LB supplemented with 2,2-dipyridyl (Dip) and ACS, Typhimurium strain 1 and 2 had higher maximum growth than Infantis-2^pESI-^ (*P*.adj < 0.05), and Typhimurium strain 1’s growth was also higher than Infantis-1^pESI-^ (*P*.adj < 0.001) (Fig. 6C). In contrast, Infantis strain 4^pESI+^ had lower growth than Infantis^pESI-^ strains (*P*.adj < 0.05) in LB supplemented with ACS, and Infantis strain 4 ^pESI+^ growth was also lower in LB supplemented with Dip and ACS than Infantis-2^pESI-^ (*P*.adj < 0.05) (Fig. 6C). Kentucky strain 2 had higher growth in LB supplemented with ACS than Infantis-2^pESI-^ (*P*.adj < 0.05), but there was no difference between Kentucky strain 1 and Infantis^pESI-^ strains (*P*.adj > 0.05). (Fig. 6B). In LB supplemented with Dip, all *Salmonella* strains experienced significantly limited growth when compared to growth in LB (*P*.adj < 0.0001) (Fig. 6D), and there were no differences (*P*.adj > 0.05) in maximum growth achieved between strains. This result supported the hypothesis that trace nutrients influenced the growth of *Salmonella* strains from this study.

### IncC plasmids harboring Ybt are present in Typhimurium genomes isolated from chicken samples

The higher growth of Typhimurium isolates compared to others in our *in vitro* experiments led us to investigate the prevalence of IncC plasmids carrying Ybt in Typhimurium isolates. To do this, we searched the NCBI database for genomes carrying an identical ~71 kb Yersinia HPI (>90% sequence coverage and >90% identity). All nine complete Typhimurium genomes harbored Ybt on an IncC plasmid. Each Typhimurium genome was classified into a different cgST, and none belonged to the same cgST as the isolates from this study (Fig. 3B). A phylogenetic tree reconstructed using the accessory genomes of the Typhimurium isolates from this study and complete publicly available genomes placed Typhimurium isolate PiLLS-234 in the same clade as two genomes in the NCBI database (CP028314 and CP022062) (Fig. S2).

To further compare the genomes of the Typhimurium isolates from this study to other isolates that have been sequenced across countries in the past three years (2021–2023), we searched the NCBI Pathogen Detection database (GenomeTrakr) (36) using the genomes from the PiLLS-234 clade (Fig. S2). Our isolates were identical to Typhimurium genomes in two SNP clusters (isolates that are within 50 SNPs of at least one other isolate in the cluster [34]). Most of the genomes in these clusters were isolated from chicken samples collected by FSIS during standard Hazard Analysis Critical Control Point sampling. All isolates in the clusters carried IncC plasmid, while *tetA* and *sul2* were present in >95% of isolates (Table S4). Additionally, Cluster PDS000180813.4 isolates tended to harbor IncC, IncX, and Col plasmids, while PDS000026712.65 isolates harbored only IncC and Col plasmids. Ninety percent of isolates in SNP cluster PDS000180813.4 (216/239) and all isolates in cluster PDS000026712.65 (40/40) harbored Ybt (Table S4). These results suggest that IncC plasmids that carry Ybt are common in Typhimurium genomes isolated from chicken samples in the United States.

## DISCUSSION

Broiler litter sampling has been shown to be a practical way to detect a *Salmonella*-infected flock at pre-harvest but suffers the limitations of traditional culture methods in terms of slow time to results. A farmer might not know the *Salmonella* status of their flock until at least 4 days after sample submission for *Salmonella* testing. In this study, we used a culture-independent qPCR method to determine the abundance of *Salmonella* tetrathionate reductase (*ttrBCD*). The qPCR assay’s sensitivity and specificity have been consistently reproduced across multiple studies over the years (28, 37–39). While overall *Salmonella* gene abundance was high, gene abundance alone was insufficient to conclude whether a litter sample would be culture positive. However, higher *Salmonella* gene abundance increased the likelihood of finding culturable salmonellae. Incorporating gene abundance into a machine learning model improved its accuracy in predicting whether a litter sample would test positive or negative for *Salmonella* culture.

Our findings align with other studies that have determined the abundance of *Salmonella* in litter. Dunn et al. (22) and Gutierrez et al. (23) utilized the most probable number (MPN) method to estimate *Salmonella* abundance in litter collected from broiler farms across five southern states. They reported a wide range in *Salmonella* load spanning from <0.45 to >280,000 MPN/g and 0.02–462.17 MPN/g. Cook et al. (40) reported that the abundance of *Salmonella ttrBCD* gene was below the limit of quantitation (LOQ) of their method (log_10_ 3.0 gene copies g^-litter^) and became detectable only after incubating the litter in buffered peptone water (BPW) for 24 h. In our study, the LOQ of our qPCR method was log_10_ 5.60 gene copies g^-litter^, and half of the litter samples (117/240) had levels above the LOQ without the need for enrichment in BPW.

The study observed differences in serotyping results using antisera, qPCR, and WGS. These discrepancies have been previously reported (41, 42). Despite the discrepancies, they did not affect our interpretation of the results. The top three serotypes (Infantis, Typhimurium, and Kentucky) found in broiler litter are consistent with the serotypes detected in raw chicken whole/quarter/half carcasses and young chicken carcasses rehang by FSIS (43, 44). Although there was low genetic diversity between sequenced isolates from each serovar, Typhimurium isolates had the largest SNP and accessory gene differences. For example, one Typhimurium isolate differed from other isolates by 51–71 SNPs and harbored other ARGs and plasmids (Fig. 3B; Fig. S2). This isolate belonged to a different core genome MLST and was grouped into a separate cluster on reconstructed phylogenetic trees.

Typhimurium and Infantis were isolated from all flocks, while 92% of Kentucky isolates were from litter sampled after the introduction of flock 4 (Fig. 2B). The sudden increase in Kentucky prevalence in flock 4 correlated with a lower *Salmonella* gene abundance and a reduction in the number of Typhimurium and Infantis isolates recovered. The average *Salmonella* gene abundance in flock 4 litter was below the limit of LOQ of our qPCR method (Fig. 1F), while total Enterobacteriaceae abundance was approximately 2-log_10_ lower in flock 4 compared to flocks 1–3 (data not shown). It is plausible that the reduction in total Enterobacteriaceae, including Typhimurium and Infantis, allowed Kentucky to proliferate and improved its detection in litter. Kentucky was isolated from litter before the placement of flock 2 and was found in the litter of flock 3 (Fig. 2B; Table S1), indicating that Kentucky was present at low levels in litter. The Kentucky strains sequenced in this study harbored fewer virulence factors compared to Typhimurium and Infantis, but the presence of a large conjugative plasmid that carried genes for copper and silver resistance, as well as the production of aerobactin siderophore, may contribute to their fitness in litter and chickens.

We do not have sufficient information to speculate on the management practices that might have caused the lower abundance of total Enterobacteriaceae in the litter of flock 4. Competition within Enterobacteriaceae significantly influences *Salmonella* population dynamics (45–49). For instance, Spragge et al. (50) demonstrated that the presence of *Escherichia coli* was crucial for mice to gain colonization resistance against *S*. Typhimurium or *Klebsiella* pneumoniae infection. The authors suggested that the ability of *E. coli* to consume nutrients that overlapped with *Salmonella* could explain this acquired resistance. Additionally, chickens vaccinated against one *Salmonella* serotype exhibit cross-protection against serovars from the same serogroup (51). Eeckhaut et al. (52) found that broilers administered a commercially available Typhimurium vaccine were also protected against Infantis colonization. Alternatively, it is possible that the fourth flock of chicks originated from a different hatchery than flocks 1–3 and carried more robust Kentucky strains compared to residual strains from earlier flocks.

All sequenced Typhimurium isolates harbored an IncC plasmid carrying ARGs for tetracycline and sulfonamide resistance (Fig. 4). However, we have no evidence to suggest that these antibiotics were administered to chickens raised on the farms studied. Despite this, their occurrence across all Typhimurium isolates in this study and chicken samples collected across the United States indicates the possibility of selection. Johnson et al. (53) demonstrated that feeding pigs a high dose of chlortetracycline increased the fecal *E. coli* population harboring IncC. Additionally, tetracyclines and sulfonamides are available to veterinarians via the Veterinary Feed Directive, and chickens with respiratory and necrotic enteritis infection are prescribed water-soluble tetracycline and sulfonamide drugs for 2–7 days (54).

Moreover, IncC plasmids also harbored an island that encoded fitness factors for yersiniabactin siderophore production. Yersiniabactin exhibits strong binding affinity for iron and copper, as well as other non-iron metals like zinc and nickel (31, 55, 56). Increasingly, *E. coli* and *Klebsiella* strains harboring Ybt have been associated with virulent and challenging-to-treat urinary and pulmonary tract infections in humans (32, 57, 58). In *S*. Infantis, Ybt expression was higher under iron-deficient conditions, and it is linked to the increased fitness of Infantis strains carrying pESI-like plasmids (59). In this study, Typhimurium strains that harbored Ybt grew better than Infantis strains that did not harbor pESI-like plasmids when exposed to LB media enriched in CuSO_4_ and limited iron (Fig. 6C). Although our experiments were not specifically designed to decipher the role of siderophores such as Ybt, they do indicate that there are differences in the growth potential of Ybt^+^ and Ybt^-^ strains when exposed to relevant environmental selection pressures. These results are consistent with previous studies showing that Enterobacteriaceae harboring Ybt can utilize copper as a nutrient source, thus increasing their fitness (56, 60, 61).

Infantis isolates found in chicken products have been suggested to be clonal based on the number of SNP differences between isolates (30, 62). Infantis strains isolated across the United States were within 31 SNPs of one another, including human clinical and chicken isolates (30). Our results support these earlier findings. In this study, Infantis isolates were within 22 SNPs of each other and harbored identical pESI-like plasmids. A comparison of SNP profiles between Infantis isolates from our study and those sequenced by Tyson et al. (30) revealed differences of 39–70 SNPs (Fig. 3A). The primary variation in the pESI-like plasmids carried by Infantis isolates occurred in region 2, which is consistent with recent findings by Li et al. (34) (Fig. 4). While all pESI-like plasmids from this study carried the expected ARGs in region 1, the frequency of two ARGs (*aph(3′)-Ia* and *dfrA14*) in region 2 was not uniform across plasmids. Therefore, we can conclude that the Infantis strains isolated from the litter samples of the broiler farm studied are similar to isolates that have been found in retail chicken meat across the United States.

Moisture is a critical factor influencing the proliferation of pathogens like *Salmonella* in litter. Our findings indicate that higher litter moisture significantly increases the likelihood of detecting culturable *Salmonella* in litter. Additionally, the Decision Tree Classifier model highlights that assessing moisture in the caked portion of litter is the most informative parameter for predicting *Salmonella* prevalence. Litter moisture is positively correlated with water activity (63), and water plays a crucial role in bacterial nutrient uptake. We found significant correlations between litter moisture, *Salmonella,* and iron.

Iron is essential for many cellular processes; however, iron can be toxic to bacterial cells at elevated levels (57). Therefore, it is crucial for bacteria to have mechanisms to regulate metal fluxes. It is possible that higher litter moisture reduces iron to levels that are less toxic and soluble (ferrous versus ferric ion) for *Salmonella* uptake. Here, litter samples that had <650 ppm iron were more likely to test positive for *Salmonella*.

*In vitro* experiments with ACS and Dip provided insights into the crosstalk between iron and copper homeostasis in *Salmonella*. When iron was limiting, the growth of all *Salmonella* strains from this study was inhibited (Fig. 6D); however, adding ACS restored growth, and Typhimurium strains that carried Yersiniabactin siderophores had the highest growth (Fig. 6C). One explanation is that excess copper can increase iron acquisition (64). It is also possible that copper chelation by Dip left some unbound iron for *Salmonella* growth. The role of Yersiniabactin is less clear, and further studies on its effects on the fitness and adaptation of *Salmonella*, as well as the mechanisms for its genetic transfer, are warranted. Together, our results support the notion that litter moisture plays an integral role in the bioavailability of trace nutrients that are needed for *Salmonella* survival in litter. Therefore, interventions aimed at minimizing leakage/drips around drinkers and maintaining dry litter could potentially limit *Salmonella* survival.

### Conclusion

In this study, we demonstrated that *Salmonella* persisted in the litter of two commercial broiler houses situated on the same farm across four consecutive flocks. We identified the serovars Typhimurium and Infantis as the predominant serotypes present. Through *in vitro* experiments, we showed that iron and copper affected the growth of Typhimurium and Infantis isolates carrying large conjugative plasmids. Utilizing machine learning, we determined that litter moisture content was the most significant environmental factor in predicting the presence of culturable *Salmonella*. Consequently, we developed a promising predictive model for *Salmonella* on farms. Collectively, our findings support the use of on-farm broiler litter sampling as a method to monitor emerging virulent pathogens in chickens.

While our study has limitations, particularly the small sample size (i.e., one farm and four flocks) and the biased selection of sequenced *Salmonella* strains, it provides valuable insights. The application of machine learning for pathogen prediction is not widely adopted, with few studies exploring its use in pre-harvest chicken production (65). The understanding of how model architecture or learning algorithms interpret various data patterns remains unclear, leading to discrepancies in identifying key factors influencing *Salmonella* prevalence. For instance, although litter pH decreased following alum application, it was the least informative parameter in the most effective model (Decision Tree Classifier), yet it was ranked second in importance in the Logistic Regression model (Table 2). The small sample size used for model training and testing could lead to overfitting, indicating the need for a larger data set for further validation.

## MATERIALS AND METHODS

### Study design

Four broiler flock cohorts were raised in succession in two co-located integrated commercial broiler houses between May 2021 and March 2022. At chick placement, each broiler house held 26,300–27,200 broilers per flock, and reused litter was the bedding material in both houses (Table S1). There was no cleanout between flocks, but mechanical conditioning was performed to remove caked litter. During the downtime between flock cohorts, a commercial litter acidifier composed of aluminum sulfate (alum) was applied to litter at a rate of 20–25 gallons/1,000 ft^2^ for ammonium control (typically <1 week before new flock placement). Whole house brooding was performed for each flock. Acidified copper sulfate was given via drinking water.

### Litter sampling

Litter was collected when the chickens were young (between 4 and 14 days old) and when they were at market age (32–38 days old). During downtime, litter was collected before alum addition (11 days after chickens from the previous flock were harvested) and after alum was applied (4–5 days before a new flock was placed). Due to logistical reasons, there was some deviation from this sampling plan for flocks 3 and 4. For flock 3, we did not collect litter samples before (house 2) and after application (house 1) and after alum application for flock 4 (houses 1 and 2). Each house was divided into four sections, and each section was divided into two subsections (Fig. S5). During sampling, nine litter grab samples were taken from each of the eight subsections, including areas underneath feeder and drinker lines (Fig. S5). All nine grab samples from each subsection were pooled into one zip-top bag. This amounted to 224 pooled litter samples. Litter samples were transported on ice to the United States National Poultry Research Center for further processing. Litter samples were processed within 24 h of collection.

### Litter processing and *Salmonella* isolation from litter

Thirty grams from each pooled litter sample was mixed with 120 mL phosphate-buffered saline and shaken with a Wrist-O-Matic Variable Speed Shaker (Boekel Scientific, Model 401000, Feasterville-Trevose, PA, USA) for 10 min. The litter eluate was used for downstream bacteriological analyses. We used a selective culture enrichment technique (a modification of the Bacteriological Analytical Manual for *Salmonella* [https://www.fda.gov/media/178914/download?attachment]) to determine the presence/absence of *Salmonella* in the litter. Buffered peptone water (Becton Dickinson, Sparks, MD, USA) was added to litter eluate (3× volume to the weight). Litter eluate (100 µL) was directly plated on both Brilliant Green Sulfur (BGS) (Becton Dickinson) and Xylose Lysine Tergitol-4 (XLT-4) (Becton Dickinson) agars. Plates were incubated for 18–24 h at 37°C. Additionally, aliquots (1 mL) of the eluate were enriched in buffered peptone water (9 mL) and incubated for ~18–24 h at 35°C. Afterward, 500 µL of the overnight BPW enrichment was transferred to Tetrathionate (TT) broth (Becton Dickinson), while 100 µL was added to Rappaport-Vassiliadis (RV) broth (Becton Dickinson). Both RV and TT broths were incubated overnight at 42°C. Thereafter, a 10 µL inoculating loop was used to spread RV and TT broth on BGS agar supplemented with 5 mg/L novobiocin agar and XLT-4 agar. Isolated colonies characteristic of *Salmonella* were streaked on triple sugar iron and lysine iron agar slants (Becton Dickinson) and incubated at 37°C for 18–24 h. One isolate per *Salmonella*-positive litter sample (*n* = 109 isolates) was stored at −80°C in lysogeny broth containing 30% glycerol.

### Determination of *Salmonella* abundance in litter via quantitative PCR

DNA was extracted from the same litter samples that were used for *Salmonella* isolation. Briefly, 250 µL of litter eluate from each litter sample was extracted using the QIAamp 96 DNA QIAcube HT Kit (Qiagen, Hilden, Germany) for QIAcube HT System (Qiagen). After the elution step of the protocol, an additional cleanup with a OneStep polymerase chain reaction (PCR) inhibitor removal kit (Zymo Research Corporation, CA, USA) was performed as per the manufacturer’s instructions. Afterward, DNA was further diluted 100-fold in 1× Tris EDTA buffer (10 mM Tris-HCl and 1 mM EDTA, pH 7.5) to reduce PCR inhibitors. qPCR amplification was performed using a CFX96 Touch real-time PCR detection system (Bio-Rad, Inc., Hercules, CA, USA). Reaction mixtures contained 1× SsoAdvanced Universal SYBR Green Supermix (Bio-Rad), 600 nM (each) primers, and 2 µL of DNA. The primers used for total Enterobacteriaceae (F:CCGTTGAAGTGAAAGACGGTC and R:AACCACTTTCTTCGCACCAGC), *Salmonella enterica* (F:CTCACCAGGAGATTACAACATGG and R:AGCTCAGACCAAAAGTGACCATC), and *S*. Infantis (F:GGTCGAGATGGGTATGTAGC and R:CAGGAGTTCCTGCGCAACCA) targeted glyceraldehyde-3-phosphate dehydrogenase A (*gapA*) (66), tetrathionate reductase (*ttrBCD*) locus (28), and uncharacterized protein (*usg*) (29), respectively. Calibration curves used for converting qPCR cycle threshold values to gene copies were determined using genomic DNA from relevant *Salmonella enterica* and *Escherichia coli* strains available in-house. The limit of quantitation for *gapA*, *ttr,* and *usg* was log_10_ 5.60 gene copies/g of litter. Relative abundance of *Salmonella* was determined using: log_10_ (*ttr* or *usg* gene copies/*gapA* gene copies)/g of litter.

### Antibiotic susceptibility testing of *Salmonella* isolates and serotyping

Broth microdilution was used to determine the susceptibility of *Salmonella* isolates (*n* = 109) to antibiotics. Susceptibility testing was performed using the Sensititre semi-automated system (Thermo Fisher Scientific, Kansas City, KS, USA) according to the manufacturer’s instructions. Bacterial suspensions equivalent to a 0.5 McFarland standard were prepared, aliquoted into a CMV4AGNF panel, and incubated at 37°C for 18 h. Minimum inhibitory concentrations were determined and categorized as resistant according to Clinical and Laboratory Standards Institute (CLSI) guidelines when available (67); otherwise, breakpoints established by the National Antimicrobial Resistance Monitoring System were used (https://www.fda.gov/media/108180/download).

For serotyping, *Salmonella* isolates were grown on tryptic soy agar slants and sent to the National Veterinary Services Laboratories (NVSL) at Ames, IA, USA, for serotyping. *Salmonella* serotyping at the NVSL is an ISO 17025 accredited test. Salmonellae were typed via classical serotyping using polyvalent and single factor antisera to determine the O and H antigens and/or via molecular typing using the xMAP *Salmonella* serotyping assay (https://www.cdc.gov/nationalsurveillance/pdfs/salmonella-serotypes-isolated-animals-and-related-sources-508.pdf).

### Whole-genome sequencing of *Salmonella* isolates and bioinformatics

Selected *Salmonella* isolates (*n* = 23) were sequenced using long-read sequencing technologies (PacBio: Pacific Biosciences, Menlo Park, CA, USA; MiniON: Oxford Nanopore Technologies, Oxford, United Kingdom) (Table S2). When it was not possible to assemble *Salmonella* genomes into complete circular chromosome and plasmid contigs using long reads only, genomes were assembled by using both short reads and long reads as a hybrid approach (Table S2). Long reads generated with PacBio were subsampled to a sequence depth of 200× before assembly was done using the Hierarchical Genome Assembly Process using default parameters (68). Isolates selected for both short read and long read sequencing were assembled using Unicycler (--hybrid) implemented through the Reads2Resistome pipeline version 0.0.2 (69). *Salmonella* serovar identification was performed using SISTR (70) via Reads2Resistome. Identification of antimicrobial resistance genes, virulence factors, and plasmid replicons was done using Abricate (https://github.com/tseemann/abricate) via Reads2Resistome using ResFinder (71), PlasmidFinder (72), BacMet (73), and Virulence Factor Database (74). Genomes were annotated using Prokka (75), the Rapid Annotation using Subsystem Technology (76), and KofamKoala (77). Single nucleotide polymorphisms were identified by CSI phylogeny (78), and maximum-likelihood phylogenetic trees were constructed by IQ-tree2 (79) with 1,000 bootstrapping.

### Determination of litter pH, moisture, and nutrients

Litter moisture was determined gravimetrically by drying approximately ~10 g (range = 3–26 g) of caked and ~5 g (range = 2–13 g) of friable litter at 107°C for 24 h. The pH of the litter eluate was measured using a portable Orion Star A series pH meter (Thermo Fisher Scientific). To determine nutrient content, litter samples were air-dried, ground to pass through a 1 mm sieve, and analyzed for chemical properties. Total nitrogen (N) and carbon (C) contents in litter were determined using a Vario Max Cube Elemental CNS Analyzer with dry combustion (Elementar Americas Inc., Mt. Laurel, NJ, USA). Total nutrient contents were measured by dry ashing 1 g sample, following the methods outlined by Isaac and Kerber (80). Samples were dry ashed in a furnace at 500°C for 4 h, digested in 1 mL of 6 M HCl for 1 h, and then digested again in 40 mL of double acid (0.0125 M H_2_SO_4_ and 0.05 M HCl) for an additional hour. The extracts underwent filtration using Whatman No. 1 filter paper. Nutrient concentrations, including phosphorus, potassium, calcium, manganese, copper, zinc, and boron, were quantified spectrophotometrically using inductively coupled plasma (Varian Analytical Instruments, Walnut Creek, CA, USA).

### Predicting *Salmonella* detection using machine learning models

All classification models in the open-access Python-based machine learning library, Scikit learn (81), were first investigated to explore their feasibility for multiple feature classification. A total of seven machine learning classifiers from the library were selected and compared to determine the best one to identify *Salmonella* presence and absence. Machine learning model performance is considerably affected by tuning hyperparameters. Hyperparameters and hyperparameter spaces for each classifier were selected based on the recommendations outlined in Scikit-learn and used to optimize the respective models. Detailed explanations of these hyperparameters can be found in the Scikit learn library (81).

Four major analyses were sequentially conducted to develop and interpret machine learning models for classifying *Salmonella* prevalence, including (i) data preprocessing; (ii) hyperparameter tuning; (iii) comparative evaluation of machine learning models; and (iv) feature importance analysis. A data set was formulated with eight input features of total Enterobacteriaceae (*gapA*), *Salmonella enterica* (*ttr*), *S*. Infantis (*usg), ttr*/*gapA, usg*/*gapA*, litter pH, caked litter moisture, and friable litter moisture and outputs of *Salmonella* prevalence, positive (1) and negative (0). A total of 224 data points were used for the machine learning classification. Those data points were split into 90% for training and 10% for testing. A random state was set to ensure all the models were trained and tested in the same data space. As the features were on different scales, to increase the robustness of the model and reduce prediction bias, all the features were normalized using equation 1. Normalization was conducted separately for training and testing data sets to avoid overfitting.


(1) $${\underline{x}}_{i}=\;\frac{x_{i}}{\sqrt{\sum_{k=1}^{n}{\left|x_{k}\right|}^{2}}},$$

where ${\underline{x}}_{i}$ is the *i*th normalized value and $x_{i}$ is the *i*th of the original value.

The hyperparameters listed in Table 1 were comparatively evaluated to determine the optimal ones for training a specific model. The grid search method was deployed to exhaustively search the optimal combination of hyperparameters for specific models. Ten-fold cross-validation was conducted during hyperparameter tuning due to the limited data samples. The purpose of cross-validation is to examine the model ability to predict unseen data with a small training data set. The 90% of training data were equally divided into 10-folds with 1-fold held out for validation and the other 9-folds for training. The procedure was iterated 10 times. The optimal hyperparameter combination for each model was determined by validation accuracy.

With the optimal hyperparameter, the seven machine learning classifiers (Table 1) were comparatively evaluated. The optimal model was selected with the highest precision (equation 2), recall (equation 3), F1 score (equation 4), and accuracy (equation 5).


(2) $$Precision=\frac{True\;positives}{True\;positives+False\;positives},$$

(3) $$Recall=\frac{True\;positives}{True\;positives+False\;negatives},$$

(4) $$F1\;score=2\times \frac{Precision\;\times Recall}{Precision+Recall},$$

(5) $$Accuracy=\frac{True\;positives+True\;negatives}{True\;positives+False\;positives+True\;negatives+False\;negatives},$$

where True positives are the number of cases in which both algorithms and ground truths reported *Salmonella* present; False positives are the number of cases in which algorithms wrongly predicted *Salmonella* present; and False negatives are the number of cases in which algorithms wrongly predicted S*almonella* present.

### Growth experiments under iron-limited and copper-enriched conditions

A swab of isolated colonies was collected from overnight cultures of eight selected *Salmonella* strains (four *S*. Infantis, two *S*. Typhimurium, and two *S*. Kentucky) (Table S3) on Blood Agar (Thermo Fisher Scientific) into 5 mL LB Miller (Becton Dickinson). A 0.5 McFarland standard was measured by Sensititre Nephelometer (approximately 1.0 × 10^7^ CFU/mL). The cultures were further diluted in LB Miller to a final concentration of 1.0 × 10^4^ CFU/mL. Four different media (i.e., LB Miller with 50 µM Dip, LB Miller with ACS, LB Miller with 50 µM Dip and 200 ppm ACS, and LB Miller) were prepared, respectively, and used for the last 10-fold dilution to a final concentration of 1.0 × 10^3^ CFU/mL per medium. ACS was prepared based on the manufacturer’s instructions (Best Veterinary Solutions Inc., Ellsworth, IA, USA). A volume of 200 µL of cultures with different media was placed into a sterile, clear 96-well culture plate in octuplets with the lid on. Using an EPOCH2 Microplate reader (Agilent Technologies, Santa Clara, CA, USA), the plate with the samples was incubated at 37°C for 48 h. The samples were orbitally shaken continuously. Readings of 600 nm optical density (OD_600_) were taken every 30 min for each well throughout the duration of the growth. Blank samples without bacterial cells were added to normalize measured OD_600_ between different media. The Gompertz model (82) was used to determine the maximum population of each well by “growthrates” (version 0.8.4) package in R (version 4.4.0).

### Identification of SNP clusters in pathogen detection database

We accessed the NCBI Pathogen Detection database (GenomeTrakr) (36) between 6 and 13 May 2024. GenomeTrakr is a “network of federal laboratories, state health and university laboratories, and 24 laboratories located outside the United States that collect and share genomic and geographic data from foodborne pathogens” (https://www.fda.gov/food/whole-genome-sequencing-wgs-program/genometrakr-network). We searched the database using the BioSample accession numbers (BioSample/GenBank accession number) of two complete Typhimurium genomes (CP028314 and CP022062) with the closest accessory genome to the Typhimurium isolates from our study. Metadata for identified SNP clusters were downloaded in CSV format (Table S4), while genome assembly files were downloaded in FASTA format.

### Statistical analyses

Bacterial gene copies were log_10_ transformed before statistical tests were performed. Fisher’s exact test was used to determine if there were significant differences in *Salmonella* prevalence and the antimicrobial resistance profile of *Salmonella* isolates across categorical variables. Mann-Whitney and Kruskal-Wallis tests were used to determine the significance level of differences in bacterial gene abundances and litter physico-chemical parameters when compared between categorical variables. The Spearman correlation test was used to determine the correlation between litter nutrients, pH, and moisture. Simple logistic regression was used to determine the relationship between the caked litter moisture and the presence/absence of *Salmonella*. Kruskal-Wallis test and Dunn’s multiple comparison test were used to determine significant differences in maximum population between strains. All statistical tests were performed using GraphPad Prism version 10.2.2 and R (version 4.4.0). Figures and graphics were generated using GraphPad Prism (version 10.2.2), R (version 4.4.0), and iTOL (version 6.0) (83).

## Data Availability

All raw short and long FASTQ reads for *Salmonella* genomes are publicly available under NCBI BioProject number PRJNA1134661.

## SUPPLEMENTAL MATERIAL

Fig. S1 aem.01388-24-s0001.pdf Heatmap of the antibiotic susceptibility testing (AST) results of *Salmonella* isolates.

Fig. S2 aem.01388-24-s0002.pdf Phylogenetic tree of the accessory genome of *Salmonella* Typhimurium, derived from the accessory gene profiles.

Fig. S3 aem.01388-24-s0003.pdf Spearman correlation analysis of litter nutrients and moisture.

Fig. S4 aem.01388-24-s0004.pdf Iron levels in litter when culturable *Salmonella* was present/absent, and total Enterobacteriaceae gene abundance when iron was lower or higher than 650 ppm in litter.

Fig. S5 aem.01388-24-s0005.pdf Mock layout of the broiler houses sampled for this study.

Supplemental legends aem.01388-24-s0006.docx Legends for all supplemental material.

Table S1 aem.01388-24-s0007.xlsx Metadata of litter samples positive for culturable *Salmonella* and nutrient levels in flock 1 litter samples.

Table S2 aem.01388-24-s0008.xlsx Metadata of *Salmonella* isolates sequenced for this study.

Table S3 aem.01388-24-s0009.xlsx Metadata of *Salmonella* isolates used for *in vitro* growth experiments.

Table S4 aem.01388-24-s0010.xlsx Metadata of *S*. Typhimurium genomes in GenomeTrakr SNP clusters.

ASM does not own the copyrights to Supplemental Material that may be linked to, or accessed through, an article. The authors have granted ASM a non-exclusive, world-wide license to publish the Supplemental Material files. Please contact the corresponding author directly for reuse.
