# Supplementary figures and images for: Broiler litter moisture and trace metals contribute to the persistence of Salmonella strains that harbor large plasmids carrying siderophores
Source: Appl Environ Microbiol. 2025 Mar 13;91(4):e01388-24. doi: 10.1128/aem.01388-24 (PMC12016502; doi:10.1128/aem.01388-24)

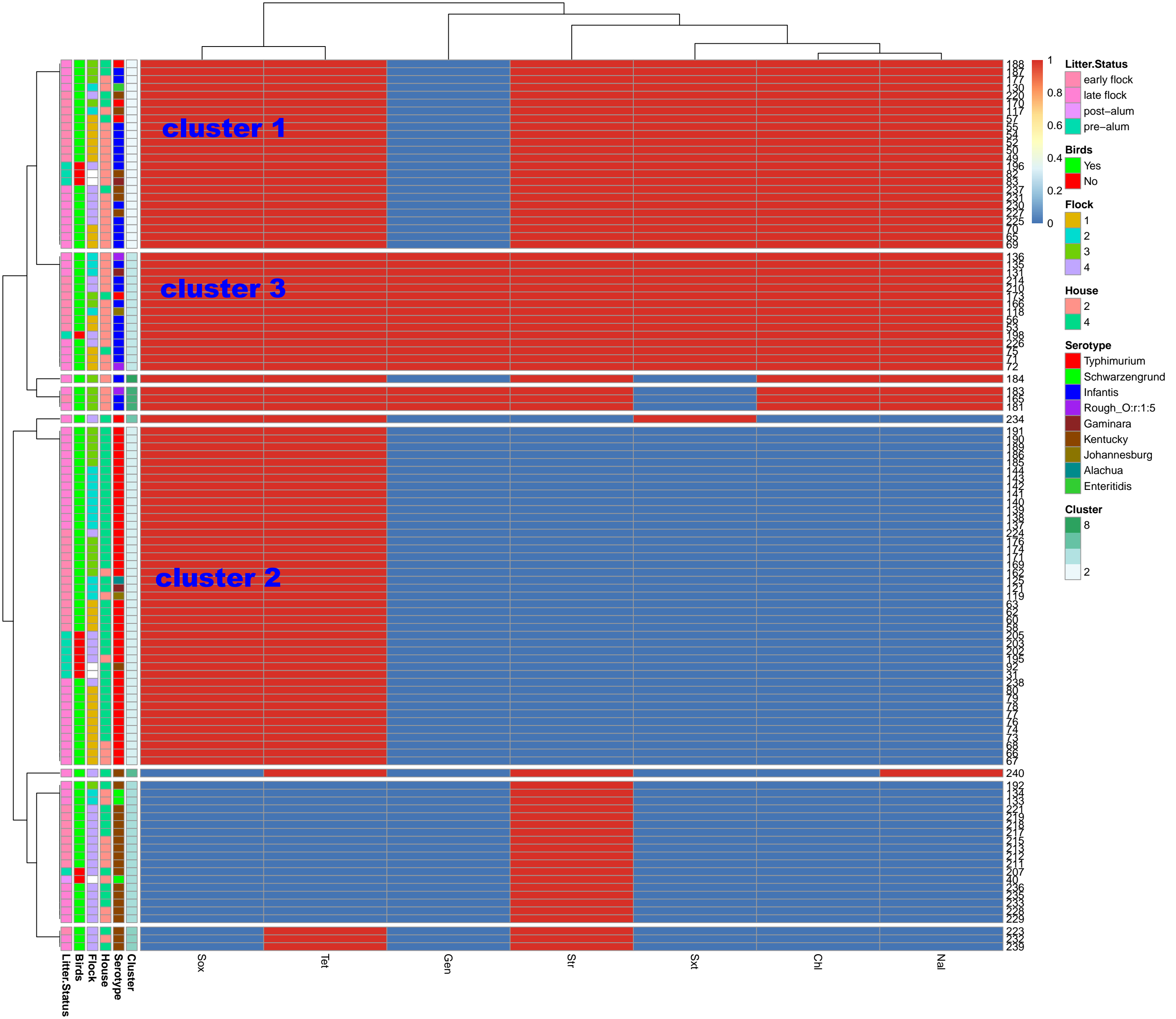

Supplement: Fig. S1 — Heatmap of the antibiotic susceptibility testing (AST) results of Salmonella isolates. [file aem.01388-24-s0001.pdf]

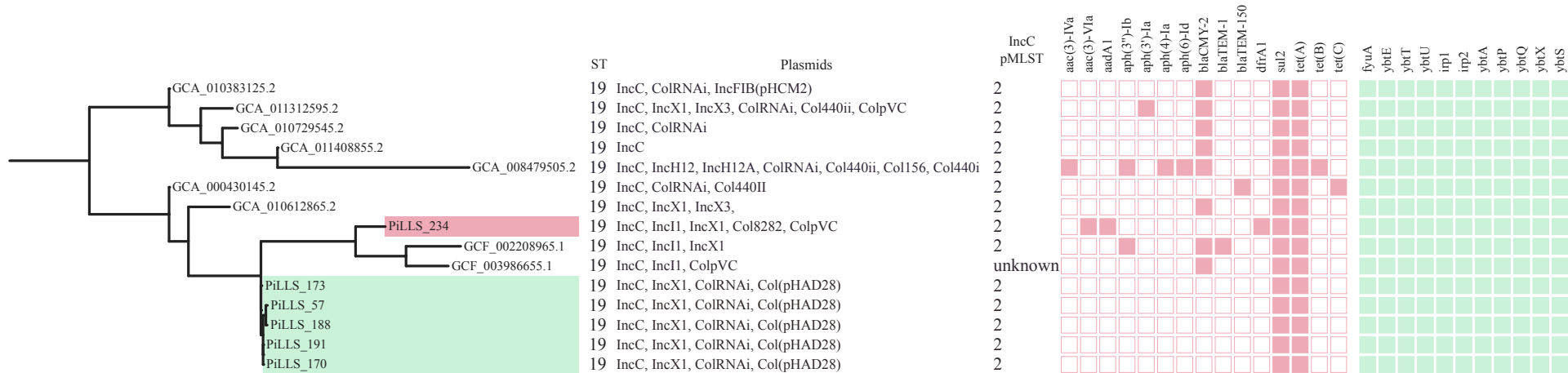

Supplement: Fig. S2 — Phylogenetic tree of the accessory genome of Salmonella Typhimurium, derived from the accessory gene profiles. [file aem.01388-24-s0002.pdf]

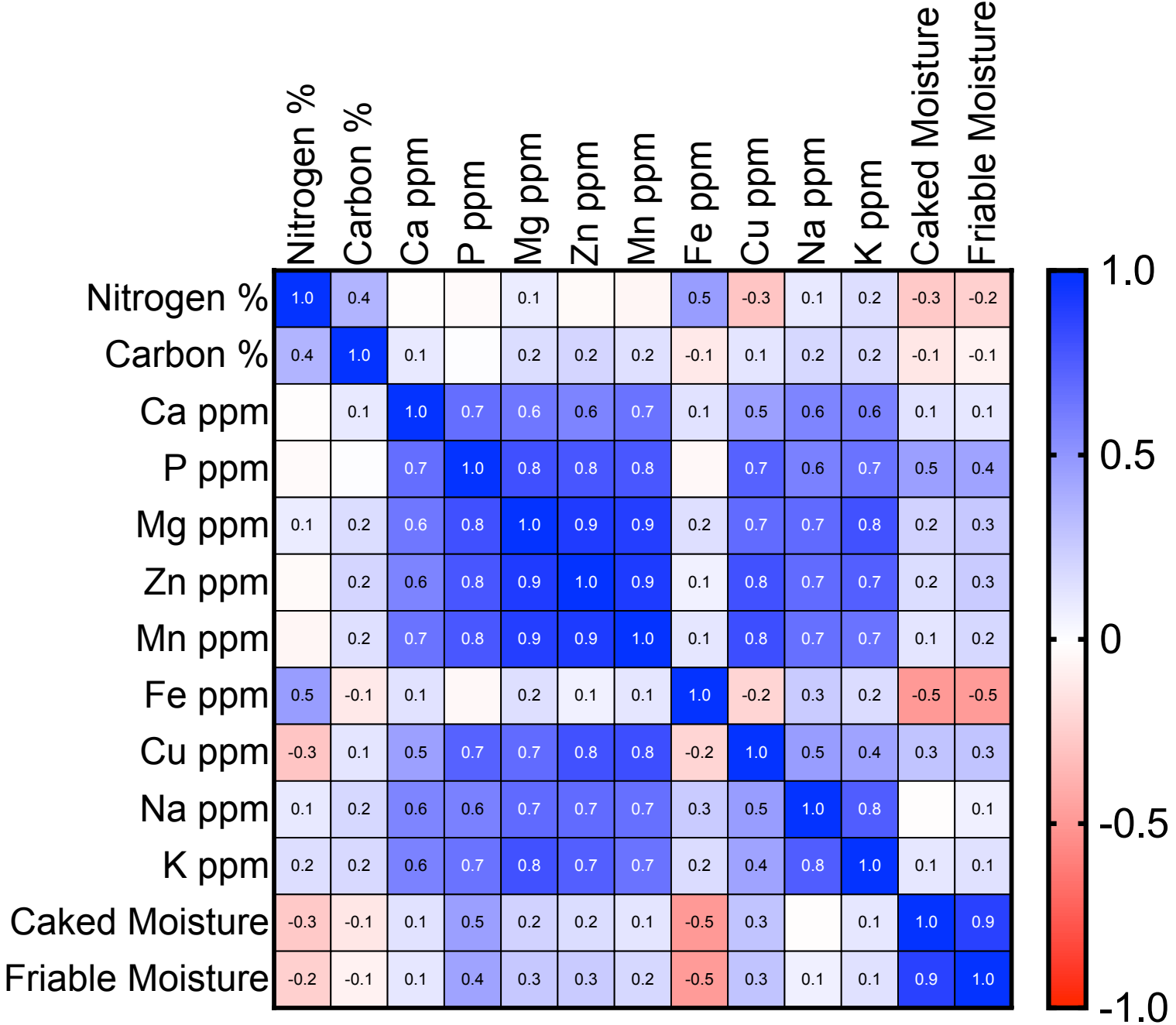

Supplement: Fig. S3 — Spearman correlation analysis of litter nutrients and moisture. [file aem.01388-24-s0003.pdf]

A

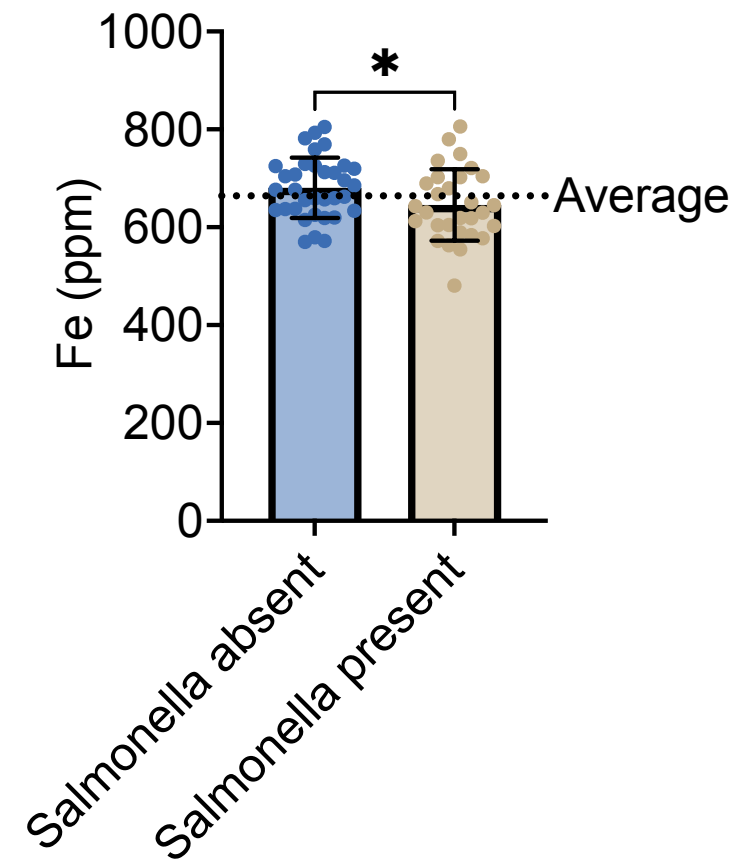

B

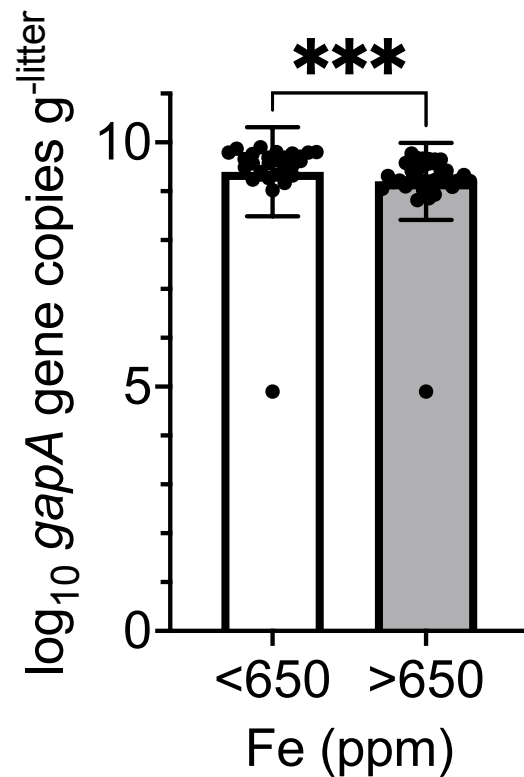

Supplement: Fig. S4 — Iron levels in litter when culturable Salmonella was present/absent, and total Enterobacteriaceae gene abundance when iron was lower or higher than 650 ppm in litter. [file aem.01388-24-s0004.pdf]

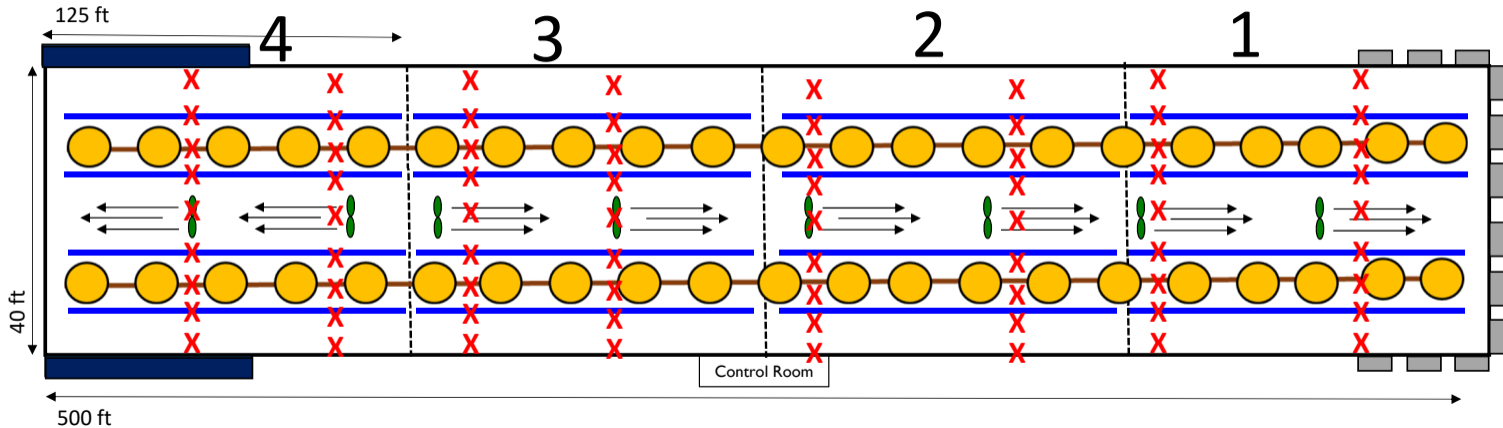

Supplement: Fig. S5 — Mock layout of the broiler houses sampled for this study. [file aem.01388-24-s0005.pdf]
