# Supplementary material for: Broiler litter moisture and trace metals contribute to the persistence of Salmonella strains that harbor large plasmids carrying siderophores
Source: Appl Environ Microbiol. 2025 Mar 13;91(4):e01388-24. doi: 10.1128/aem.01388-24 (PMC12016502; doi:10.1128/aem.01388-24)
Supplement: Supplemental legends — Legends for all supplemental material. [file aem.01388-24-s0006.docx]

**Supplemental Material Legends and Captions**

Fig. S1 Heatmap of the antibiotic susceptibility testing (AST) results of *Salmonella* isolates. Each row is one isolate tested for susceptibility against the CMV4AGNF panel. Chl, chloramphenicol; Gen, gentamicin; Nal, nalidixic acid; Str, streptomycin, Sox, sulfisoxazole; Tet, tetracycline; Sxt, trimethoprim-sulfamethoxazole. Clusters 1 and 3 include non-Infantis isolates that had similar AST as Infantis isolates, while cluster 2 are composed of mainly Typhimurium isolates.

Fig. S2 The phylogenetic tree of the accessory genome of *Salmonella* Typhimurium, derived from the accessory gene profiles. Adjacent to the tips of the tree are (i) MLST sequence types (ST), (ii) the plasmid profiles of each genome, (iii) the plasmid MLST sequence types of IncC plasmid, and (iv) heatmaps displaying the presence of antimicrobial resistance genes and selected virulence factors. Outgroups are Typhimurium genomes found on NCBI that harbored IncC plasmids that were identical to ones in this study.

Fig. S3. Spearman correlation analysis of litter nutrients and moisture. Numbers in square boxes are correlation coefficients.

Fig. S4. (A) Iron levels in litter when culturable Salmonella was present/absent and (B) total Enterobacteriaceae gene abundance when iron was lower or higher than 650 ppm in litter. Mann-Whitney test; *p < 0.05, ***P< 0.001.

Fig. S5. Mock layout of the broiler houses sampled for this study. Dimensions are not exact. Red X symbol denotes locations where grab samples were collected in each section. Solid blue horizontal line shows the drinker line, while yellow oval shapes show the drinkers.

Table S1. Metadata of litter samples positive for culturable *Salmonella* and nutrients levels in flock 1 litter samples.

Table S2. Metadata of *Salmonella* isolates sequenced for this study.

Table S3. Metadata of *Salmonella* isolates used for *in vitro* growth experiments.

Table S4. Metadata of *S*. Typhimurium genomes in GenomeTrakr SNP clusters.
